# Weighted-Incidence Syndromic Combination Antibiogram (WISCA) to Support Empirical Antibiotic Therapy Decisions in Infected Ischemic Leg Ulcers—A Feasibility Study

**DOI:** 10.3390/jcm13206219

**Published:** 2024-10-18

**Authors:** Jonas Salm, Franziska Ikker, Elias Noory, Ulrich Beschorner, Tobias Siegfried Kramer, Dirk Westermann, Thomas Zeller

**Affiliations:** 1 Department of Cardiology and Angiology, University Heart Center Freiburg—Bad Krozingen, Medical Center—University of Freiburg, Faculty of Medicine, University of Freiburg, 79085 Freiburg im Breisgau, Germanyelias.noory@uniklinik-freiburg.de (E.N.); ulrich.beschorner@uniklinik-freiburg.de (U.B.); dirk.westermann@uniklinik-freiburg.de (D.W.); thomas.zeller@uniklinik-freiburg.de (T.Z.); 2 Charité—Universitätsmedizin Berlin, Corporate Member of Freie Universität Berlin and Humboldt-Universität zu Berlin, Institute for Hygiene and Environmental Medicine, 12203 Berlin, Germany; t.kramer@ladr.de; 3 LADR der Laborverbund Dr. Kramer & Kollegen, 21502 Geesthacht, Germany

**Keywords:** chronic limb-threatening ischemia, ischemic foot infection, antibiotics, antimicrobial resistance, peripheral artery occlusive disease

## Abstract

**Objective**: Patients with peripheral artery occlusive disease (PAD) are at risk of developing foot ulcers, which can subsequently lead to foot infections and an increased risk of amputation. In cases of severe ischemic foot infections (IFIs), the empirical use of antibiotics can be limb-saving. However, there is currently no evidence-based guidance on the choice of empirical antibiotic therapy for IFI. **Methods and Design:** This retrospective single-center cohort study included 216 hospitalized patients with severe IFI undergoing endovascular revascularization. Weighted-Incidence Syndromic Combination Antibiograms (WISCAs) were calculated to guide empirical antibiotic choice. **Results:** The two most common causative pathogens for IFI were *S. aureus* and *P. aeruginosa*, with frequencies of 19.8% and 6.1%, respectively. The calculation of WISCAs revealed a low empirical coverage of amoxicillin (AMX) or clindamycin (CLN) with 21.6% and 27.7%, respectively. The empirical coverage of amoxicillin/clavulanic-acid (AMC), trimethoprim/sulfmethoxazole (SXT), and ciprofloxacin (CIP) was 50.6%, 53.1%, and 55.4%, respectively. Piperacillin/tazobactam (PT) exhibited the highest empirical coverage, with 82.5% as calculated by WISCAs. The calculated WISCAs did not significantly alter when stratified by the clinical characteristics of the patients. **Conclusions:** The empirical antibiotic coverage of CLN and AMX was low. SXT represents a promising empirical alternative in the case of IFI, irrespective of comorbidities and the WIfI score. WISCAs can assist in the decision-making process regarding empirical antibiotic therapy choices in cases of IFI.

## 1. Introduction

Peripheral artery occlusive disease (PAD) continues to be an important cause for peripheral ulcers and necrosis [1]. Patients presenting with ischemic wounds are considered to have a chronic limb-threatening ischemia (CLTI), which is associated with a high mortality and amputation rate [2,3]. Patients with PAD and peripheral wounds are at risk of developing forefoot infections, which, in the following, are defined as ischemic foot infections (IFI) [4]. The presence of IFI even increases the risk of amputation [3,5]. In addition to the significance of prompt treatment for ischemia, it is imperative to administer an efficacious antibiotic regimen in cases of severe IFI. Nevertheless, there is a paucity of evidence regarding the use of antibiotics in patients with IFI. Antibiotic treatment for IFI is often empirical, orally administered, and, in consideration of the guidelines, potentially broad spectrum [6,7]. In accordance with the recommendations for the treatment of diabetic foot infections (DFI), the choice of empirical antibiotic therapy should be based on the local likelihood of causative pathogens [8]. Local antibiograms are mainly described by cumulative hospital antibiograms [9,10]. Cumulative antibiograms, however, only report the sensitivity of individual bacterial species to certain antibiotics and the potential frequencies of causative bacteria in certain diseases. Consequently, the approach of a cumulative antibiogram is counterfactual to the approach of a clinician who is administering empirical antibiotic drugs. In contrast to the cumulative antibiogram, which commences with the isolation of the pathogen, the treating clinician is not aware of the isolated pathogen but of the patient’s characteristics and presenting disease. Based on these limitations the Weighted-Incidence Syndromic Combination Antibiogram (WISCA) was developed by Hebert et al. [11]. The WISCA is a tool that was developed to guide empirical antibiotic choices by considering the frequencies of causative pathogens, their antimicrobial resistance, and their distribution stratified by patient characteristics. The objective of the study was to identify efficacious empirical antibiotic regimes for patients with IFI using the WISCA approach.

## 2. Methods

### 2.1. Study Design

A retrospective analysis was conducted on data from IFI patients between 2012 and 2021. The dataset was selected from a prospectively maintained database. The data included microbiological diagnostics of infected wound specimens from all enrolled patients (*n* = 712). The microbiological diagnostics and data storage were performed by the medical laboratories of the private laboratory group Medical Care Center (MVZ) Clotten, Freiburg, Germany. The HyBASE hygiene management system was used to extract the microbiological datasets, which were then stored as pseudonymized Excel files.

### 2.2. Study Participants

The study included only patients with PAD Rutherford categories 5 and 6. In order to be eligible for inclusion, patients had to present with an infected arterial leg ulcer and necrotic wounds. Peripheral artery disease was angiographically confirmed. To ensure that only patients with relevant foot infections were included, we selected only patients with white blood cell (WBC) counts above 10,000/μL. All patients with ulcers were photo documented. Upon admission, each patient underwent a series of non-invasive diagnostic procedures, including ankle brachial index (ABI) measurements and duplex sonography. This was followed by angiography and, if necessary, endovascular intervention. The wounds of each patient were graded using the wound, ischemia, foot infections (WIfI) classification, as proposed by the Society for Vascular Surgery [5]. This was conducted by an independent observer (FI). Patients with a Foot Infection Score of less than or equal to one were excluded from the study. For each study participant, we had complete microbiological test results for their corresponding wound specimens. The microbiological results were assessed by an independent expert in clinical microbiology (TSK) and classified into three categories: (i) pathogen, (ii) potential pathogen, and (iii) special circumstances/commensal. In cases where more than three pathogens were identified, all pathogens classified as special circumstances/commensal were ultimately excluded. Given that a patient may be infected with multiple bacteria, the number of patients is less than the number of bacterial isolates. Systemic inflammatory response syndrome (SIRS) was defined as body temperature above 38 °C and leucocyte count >12,000/μL.

### 2.3. Ethics

The study received approval from the Ethical Board of the University of Freiburg, Germany (proposal number: 22-1238-S1-retro). The ethics committee waived the requirement for participant informed consent.

### 2.4. Specimens and Microbiology

Wound specimens and swabs were collected following a standardized protocol, as previously described [12]. Wound specimens were collected on admission before endovascular treatment. Pathogen identification and antimicrobial susceptibility testing (AST) were performed using automated systems, including MALDI-TOF, Vitek2 (bioMérieux, Marcy-l’Étoile, France), disc diffusion, and microbroth dilution. Susceptibility testing was conducted for antibiotics typically administered in IFI. The antimicrobial agents used in this study were amoxicillin/clavulanic acid (AMC), piperacillin/tazobactam (TZP), clindamycin (CLN), ciprofloxacin (CIP), trimethoprim/sulfamethoxazole (SXT), and amoxicillin (AMX). The results were interpreted according to the guidelines provided by the European Committee on Antimicrobial Susceptibility Testing (EUCAST) [13].

### 2.5. Weighted-Incidence Syndromic Combination Antibiogram

**Figure 1:**
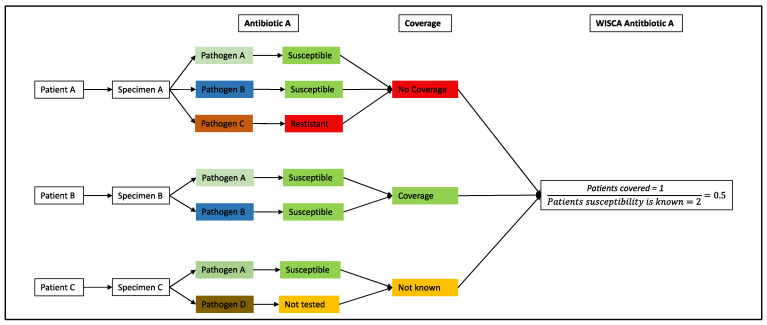
Schematic of WISCA calculation for a specific antibiotic called “A”. The numerator is the number of patients in which all recovered organisms for their infection would be covered by this specific antibiotic regime. In the schematic labeled as “Coverage” vs. “No Coverage”, the denominator are all patients in which susceptibility testing for all recovered pathogens for this specific antibiotic regime is known.

Weighted-Incidence Syndromic Combination Antibiograms (WISCAs) were calculated as follows: The numerator is the number of patients in which all the recovered organisms for their infection would be covered by a specific antibiotic regime, and the denominator were all patients in which the susceptibility for all recovered pathogens for this specific antibiotic regime is known. A patient was defined as covered by an antibiotic regime only if all pathogens identified per sample were covered. For the calculation of WISCAs, only pathogens with a total frequency of two percent were considered in an attempt to minimize the bias due to identified commensals. For further illustration, see Figure 1.

### 2.6. Statistical Analysis

The patient characteristics were analyzed in a descriptive manner, providing an overview of the data. The data were presented as means with standard deviations (SD) for continuous variables. Categorical variables were presented as counts with percentages or median with interquartile range, as appropriate. Then, 95% confidence intervals (CIs) for proportions were calculated using the Clopper–Pearson method. All statistical analyses were conducted using the free statistical computing and graphical software R (R 4.0.3; R Foundation, Vienna, Austria). The level of statistical significance was set at α = 0.05.

## 3. Results

### 3.1. Study Population

A total of 216 patients were included in the study. Of these, 76 were female (35.2%). The mean age was 75.7 years (SD = 10.6). The median WIfI categories for wound, ischemia, and foot infection were 2, 3, and 3, respectively, with a mean WBC count of 15,453.2/μL. A total of 29 patients developed a systemic inflammatory response syndrome (SIRS) following endovascular treatment, representing 13.6% of the study population (Table 1).

### 3.2. Pathogen Distribution

**Figure 2:**
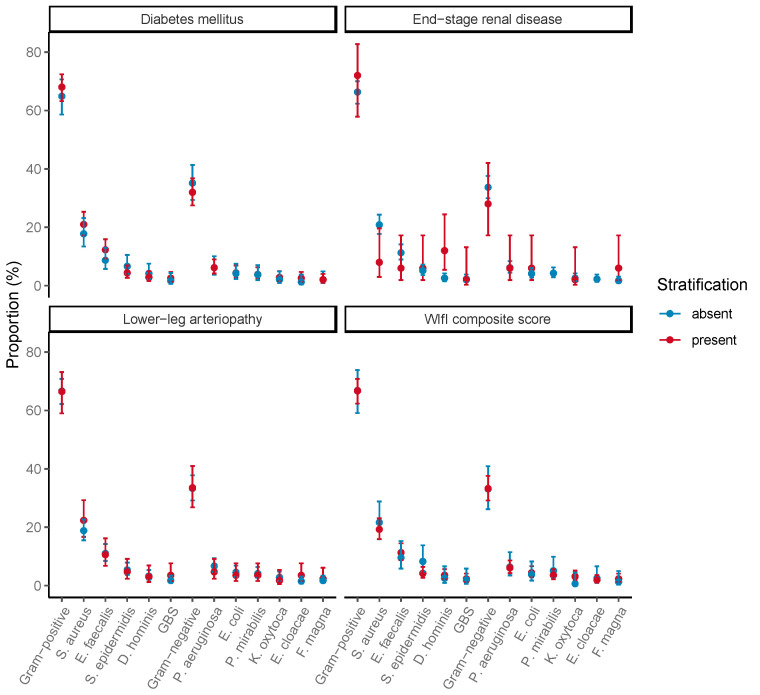
Distribution of pathogens in patients with peripheral artery disease and infected ischemic leg ulcers. The results are stratified by the clinical characteristics: diabetes mellitus, end-stage renal disease, lower-leg arteriopathy, and WIfI composite score. The frequency of bacteria is listed on the y-axes in percent, and the bacteria are listed on the x-axes. Error bars illustrate 95% confidence intervals. With regard to the WIfI composite score, the stratification is not labeled into “absent” and “present” but rather into WIfI composite score ≤ 2 vs. score > 3. WIfI: wound, ischemia, and foot infection score according to the Society of Vascular Surgery. GBS: group B *streptococci*.

The total number of identified bacteria was found to be 635. The most prevalent bacteria were Gram-positive (GPB), representing 66.8% of the total, while Gram-negative (GNB) accounted for 33.2%. *Staphylococcus aureus* was the most common bacterium, representing 19.8% of the total, while *Pseudomonas aeruginosa* was the most prevalent GNB, at 6.1% (Table 2). There was no significant difference in the frequencies of pathogens when stratified by clinical characteristics (Figure 2).

### 3.3. Weighted-Incidence Syndromic Combination Antibiogram

**Figure 3:**
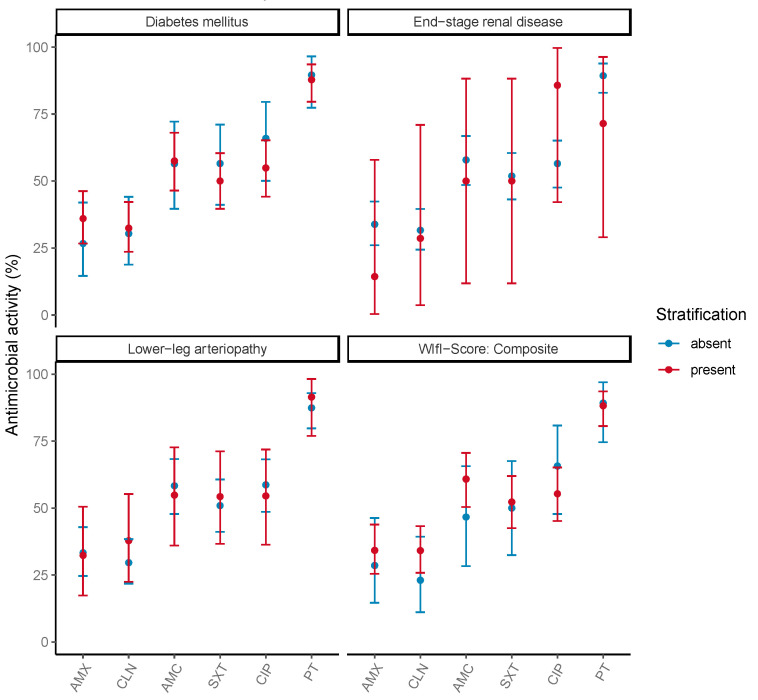
Weighted-Incidence Syndromic Combination Antibiogram (WISCA) for patients with peripheral artery disease and infected ischemic leg ulcers. The results are stratified by the clinical characteristics: diabetes mellitus, end-stage renal disease, lower-leg arteriopathy, and WIfI composite score. The proportion of antimicrobial coverage is listed on the y-axes in percent, and the tested antibiotics are listed on the x-axes. Error bars illustrate 95% confidence intervals. With regard to the WIfI composite score, the stratification is not labeled into “absent” and “present” but rather into WIfI composite score ≤ 2 vs. score > 3. WIfI: wound, ischemia, and foot infection score according to the Society of Vascular Surgery. AMX: amoxicillin, CLN: clindamycin, AMC: amoxicillin/clavulanic acid, SXT: trimethoprim/sulfamethoxazole, CIP: ciprofloxacin, PT: piperacillin/tazobactam.

AMX and CLN exhibited the lowest empirical activity, with a coverage of 21.6 and 27.7% of patients, respectively. In contrast, piperacillin/tazobactam demonstrated the highest empirical activity, with 82.5% (73.8–89.3). Empirical activity was 50.6%, 53.1%, and 55.4% in the case of treatment with AMC, SXT, and CIP, respectively (Table 3). Stratification by clinical characteristics did not significantly alter the calculated empirical susceptibilities (Figure 3).

## 4. Discussion

The data indicate that the likelihood of antibiotic coverage is not dependent on admission-known clinical characteristics. Monotherapy with AMX or CLN has low empirical susceptibility. Further, AMC, SXT, and CIP are equally active with 50.6%, 53.1%, and 55.4%, respectively. PT covered 82.5% of patients empirically.

In patients with DFI and the need for empirical antibiotic therapy, current guidelines recommend a list of antibiotics, including clindamycin, linezolid, daptomycin, and fluoroquinolones, despite the consideration of local resistance. For patients with mild DFI who reside in North America or Western Europe, the guidelines recommend even targeting empiric antibiotic therapy at aerobic GPB [8]. However, the aforementioned recommendations for DFI cannot be extrapolated to IFI patients. In patients with IFI, the distribution of pathogens differs from that observed in DFI patients. In particular, there is a higher prevalence of GNB [12,14,15]. It is possible to identify reasonable antibiotic recommendations by considering the differences in pathogen distribution and the novel approach of WISCAs. AMC, SXT, and CIP are empirical treatments for IFI that have sufficient empirical coverage. In consideration of antibiotic stewardship and the need to lower the use of broad-spectrum antibiotics, we would not recommend the empirical use of AMC [16]. Further, due to the higher age of patients with PAD (study group mean age: 75.7 years), we would not recommend CIP as empirical therapy either due to a higher prevalence of undesirable drug effects such as tendon rupture [17]. In contrast, SXT has few side effects and a high empirical coverage of 53.1%. It is important to note that in the case of the oral anticoagulation of PAD patients with vitamin K antagonists (VKAs), the international normalized ratio (INR) should be observed more strictly due to the longer metabolization of VKAs in the presence of SXT [18]. In cases of severe IFIs, our data suggest the use of PT as empirical therapy, with a coverage of 82.5%.

It is important to note that the results of our study are based on IFIs with a foot infection score of at least two. This approach ensured that only relevant forefoot infections, which appear macroscopically infected, were included. This was determined by a mean WBC count of 15,453.2/μL in patients with IFI upon admission. Furthermore, only pathogens that were rated as pathogen or potential pathogen were included in the analyses. Ultimately, our objective was to identify the pathogens that were most likely to be causative or potential causative pathogens in our dataset. Nevertheless, as a WISCA is calculated as a division with all patients in which all the recovered organisms for their infection would be covered by a specific antibiotic regime as the numerator and all patients in which the susceptibility for all recovered pathogens for this specific antibiotic regime is known as the denominator [11], potential non-causative pathogens that are not covered by an antibiotic regime ultimately bias the estimates towards lower empirical coverage.

The study is subject to several limitations. Primarily, it is a retrospective data analysis of prospectively obtained data. Although the data themselves were prospectively obtained, the database was designed primarily for interventional analysis rather than for foot-infection-associated analysis. Secondly, it should be noted that the study only contains data from a single center. As a result, it is not possible to generalize the results. This is particularly important given that pathogen and antimicrobial resistance (AMR) patterns can differ between regions [19]. Next, the invasiveness of a detected pathogens is of key importance to determine an isolate’s role in infection. The sampling strategy also included swabs, potentially overestimating the importance of detected isolates. However, we included only patients with a manifest infection, thereby reducing the role of potential contaminants and colonizing species.

## 5. Conclusions

The empirical antibiotic coverage of clindamycin and amoxicillin is low. Trimethoprim/sulfamethoxazole represents a promising empirical alternative in the case of IFI, irrespective of comorbidities and the WIfI score. WISCAs can assist in the decision-making process regarding empirical antibiotic therapy choices in cases of severe IFI.

## Figures and Tables

**Table 1:** Study population.

	Overall (N = 216)
Age (years)	
Mean (SD)	75.7 (10.6)
Sex	
Female (*n* (%))	76 (35.2)
Comorbidities (*n* (%))	
Arterial hypertension	194 (89.8)
Hypercholesterolemia	163 (75.8)
Diabetes mellitus	135 (62.8)
Nicotine consumption	89 (41.4)
End-stage renal disease	16 (7.4)
Intervened vessels (*n* (%))	
Lower leg arteries ^1^	147 (68.1)
Femoropopliteal arteries	111 (51.4)
External iliac artery	18 (8.4)
Deep femoral artery	19 (8.8)
Common iliac artery	8 (3.7)
WIfI classification ^2^ (median (IQR))	
Wound	2.0 (2.0, 2.0)
Ischemia	3.0 (1.8, 3.0)
Foot infection	3.0 (3.0, 3.0)
Limb and survival outcomes	
Minor amputation	67 (31.2)
Major amputation	30 (14.0)
Periinterventional death ^3^	13 (6.0)
Systemic inflammation	29 (13.6)
Wound localization (*n* (%))	
Right	110 (50.9)
Left	106 (49.1)
Toes	100 (46.3)
Foot	87 (40.3)
Lower limb	29 (13.4)
Laboratory parameters (mean (SD))	
CRP ^4^ (mg/dL)	6.8 (8.1)
WBC ^5^ (*n*/µL)	15,453.2 (14,084.2)
HbA1c (%)	6.7 (1.7)
Microbiology	
Polymicrobial (*n* (%))	154 (71.3)
N bacterial isolates (median (IQR))	2.0 (1.0, 4.0)

^1^ Lower leg arteries: tibioperoneal trunk, anterior tibial artery, posterior tibial artery, peroneal artery; ^2^ wound, ischemia, and foot infection classification according to the society of vascular surgery; ^3^ death 30 days after the procedure; ^4^ C-reactive protein; ^5^ white blood cell count.

**Table 2:** Pathogen distribution in infected ischemic leg ulcers, 2012–2020.

Pathogen (*n* = 635)	*n*	Percent (%)	95% CI ^1^
Gram-positive	424	66.8	63.0; 70.3
* Staphylococcus aureus*	126	19.8	16.9; 23.1
*Enterococcus faecalis*	69	10.9	8.7; 13.5
*Staphylococcus epidermidis*	33	5.2	3.7; 7.2
*Dermabacter hominis*	21	3.3	2.2; 5.0
GBS ^2^	14	2.2	1.3; 3.7
Gram-negative	211	33.2	29.7; 37.0
*Pseudomonas aeruginosa*	39	6.1	4.5; 8.3
*Escherichia coli*	27	4.3	2.9; 6.1
*Proteus mirabilis*	25	3.9	2.7; 5.8
*Klebsiella oxycota*	16	2.5	1.5; 4.1
*Enterobacter cloacae*	13	2.0	1.2; 3.5
Obligate anaerobic			
*Finegoldia magna*	13	2.0	1.2; 3.3

^1^ 95% confidence interval; ^2^ group B streptococci.

**Table 3:** Weighted-Incidence Syndromic Combination Antibiogram (WISCA) for patients with peripheral artery disease and infected ischemic leg ulcers, 2012–2021.

Antibiotic	N Covered Patients ^1^	N Patients ^2^	Percent (%)	95% CI ^3^
AMX	21	97	21.6	13.9; 31.2
CLN	33	119	27.7	29.9; 36.7
AMC	41	81	50.6	39.3; 61.9
SXT	52	98	53.1	42.7; 63.2
CIP	51	92	55.4	44.7; 65.8
PT	85	103	82.5	73.8; 89.3

^1^ Only if all isolated pathogens per patient were covered by antibiotic regime; ^2^ all patients for which microbiological test results for all isolated pathogens were available; ^3^ 95% confidence interval; AMX: amoxicillin; CLN: clindamycin; AMC: amoxicillin/clavulanic acid; SXT: trimethoprim/sulfamethoxazole; CIP: ciprofloxacin; PT: piperacillin/tazobactam.

## Data Availability

Data is contained within the article.

## References

[1] Mensah G.A., Fuster V., Murray C.J., Roth G.A., Global Burden of Cardiovascular Diseases and Risks Collaborators (2023). Global Burden of Cardiovascular Diseases and Risks, 1990–2022. J. Am. Coll. Cardiol..

[2] Reinecke H., Unrath M., Freisinger E., Bunzemeier H., Meyborg M., Lüders F., Gebauer K., Roeder N., Berger K., Malyar N.M. (2015). Peripheral Arterial Disease and Critical Limb Ischaemia: Still Poor Outcomes and Lack of Guideline Adherence. Eur. Heart J..

[3] Darling J.D., McCallum J.C., Soden P.A., Guzman R.J., Wyers M.C., Hamdan A.D., Verhagen H.J., Schermerhorn M.L. (2017). Predictive Ability of the Society for Vascular Surgery Wound, Ischemia, and Foot Infection (WIfI) Classification System after First-Time Lower Extremity Revascularizations. J. Vasc. Surg..

[4] Martelli E., Zamboni M., Sotgiu G., Saderi L., Federici M., Sangiorgi G.M., Puci M.V., Martelli A.R., Messina T., Frigatti P. (2023). Sex-Related Differences and Factors Associated with Peri-Procedural and 1 Year Mortality in Chronic Limb-Threatening Ischemia Patients from the CLIMATE Italian Registry. J. Pers. Med..

[5] Mills J.L., Conte M.S., Armstrong D.G., Pomposelli F.B., Schanzer A., Sidawy A.N., Andros G. (2014). The Society for Vascular Surgery Lower Extremity Threatened Limb Classification System: Risk Stratification Based on Wound, Ischemia, and Foot Infection (WIfI). J. Vasc. Surg..

[6] Aboyans V., Ricco J.-B., Bartelink M.-L.E.L., Björck M., Brodmann M., Cohnert T., Collet J.-P., Czerny M., De Carlo M., Debus S. (2018). 2017 ESC Guidelines on the Diagnosis and Treatment of Peripheral Arterial Diseases, in Collaboration with the European Society for Vascular Surgery (ESVS): Document Covering Atherosclerotic Disease of Extracranial Carotid and Vertebral, Mesenteric, Renal. Eur. Heart J..

[7] Gerhard-Herman M.D., Gornik H.L., Barrett C., Barshes N.R., Corriere M.A., Drachman D.E., Fleisher L.A., Fowkes F.G.R., Hamburg N.M., Kinlay S. (2017). 2016 AHA/ACC Guideline on the Management of Patients with Lower Extremity Peripheral Artery Disease: A Report of the American College of Cardiology/American Heart Association Task Force on Clinical Practice Guidelines. Circulation.

[8] Senneville É., Albalawi Z., van Asten S.A., Abbas Z.G., Allison G., Aragón-Sánchez J., Embil J.M., Lavery L.A., Alhasan M., Oz O. (2024). IWGDF/IDSA Guidelines on the Diagnosis and Treatment of Diabetes-Related Foot Infections (IWGDF/IDSA 2023). Clin. Infect. Dis..

[9] Macdonald K.E., Boeckh S., Stacey H.J., Jones J.D. (2021). The Microbiology of Diabetic Foot Infections: A Meta-Analysis. BMC Infect. Dis..

[10] Wu W.-X., Liu D., Wang Y.-W., Wang C., Yang C., Liu X.-Z., Mai L.-F., Ren M., Yan L. (2017). Empirical Antibiotic Treatment in Diabetic Foot Infection: A Study Focusing on the Culture and Antibiotic Sensitivity in a Population from Southern China. Int. J. Low. Extrem. Wounds.

[11] Hebert C., Ridgway J., Vekhter B., Brown E.C., Weber S.G., Robicsek A. (2012). Demonstration of the Weighted-Incidence Syndromic Combination Antibiogram: An Empiric Prescribing Decision Aid. Infect. Control Hosp. Epidemiol..

[12] Salm J., Böhme T., Noory E., Beschorner U., Kramer T.S., Westermann D., Zeller T. (2023). Arterial Leg Ulcers—Bacterial Patterns, Antimicrobial Resistance and Clinical Characteristics, a Retrospective Single-Centre Cohort, 2012–2021. PLoS ONE.

[13] The European Committee on Antimicrobial Susceptibility Testing Breakpoint Tables for Interpretation of MICs and Zone Diameters. http://www.eucast.org.

[14] Lipsky B.A., Pecoraro R.E., Larson S.A., Hanley M.E., Ahroni J.H. (1990). Outpatient Management of Uncomplicated Lower-Extremity Infections in Diabetic Patients. Arch. Intern. Med..

[15] Lipsky B.A., Berendt A.R., Deery H.G., Embil J.M., Joseph W.S., Karchmer A.W., LeFrock J.L., Lew D.P., Mader J.T., Norden C. (2004). Diagnosis and Treatment of Diabetic Foot Infections. Clin. Infect. Dis..

[16] Murray C.J.L., Ikuta K.S., Sharara F., Swetschinski L., Robles Aguilar G., Gray A., Han C., Bisignano C., Rao P., Wool E. (2022). Global Burden of Bacterial Antimicrobial Resistance in 2019: A Systematic Analysis. Lancet.

[17] O’Donnell J.A., Gelone S.P. (2000). FLUOROQUINOLONES. Infect. Dis. Clin. N. Am..

[18] Visser L.E., Penning-van Bees F.J.A., Kasbergen A.A.H., De Smet P.A.G.M., Vulto A.G., Hofman A., Stricker B.H.C. (2002). Overanticoagulation Associated with Combined Use of Antibacterial Drugs and Acenocoumarol or Phenprocoumon Anticoagulants. Thromb. Haemost..

[19] Hatipoglu M., Mutluoglu M., Turhan V., Uzun G., Lipsky B.A., Sevim E., Demiraslan H., Eryilmaz E., Ozuguz C., Memis A. (2016). Causative Pathogens and Antibiotic Resistance in Diabetic Foot Infections: A Prospective Multi-Center Study. J. Diabetes Complicat..

